# EcoFAB 3.0: a sterile system for studying sorghum that replicates previous field and greenhouse observations

**DOI:** 10.3389/fpls.2024.1440728

**Published:** 2024-10-07

**Authors:** Kshitiz Gupta, Yang Tian, Aymerick Eudes, Henrik V. Scheller, Anup K. Singh, Paul D. Adams, Peter F. Andeer, Trent R. Northen

**Affiliations:** ^1^ Technology Division, Joint BioEnergy Institute, Emeryville, CA, United States; ^2^ Engineering Directorate, Lawrence Livermore National Laboratory, Livermore, CA, United States; ^3^ Feedstocks Division, Joint BioEnergy Institute, Emeryville, CA, United States; ^4^ Environmental Genomics and Systems Biology, Lawrence Berkeley National Laboratory, Berkeley, CA, United States

**Keywords:** fabricated ecosystems, sorghum, plant-microbe interactions, sustainable agriculture, bioenergy crops

## Abstract

**Introduction:**

Studying plant-microbe interactions is one of the key elements in understanding the path to sustainable agricultural practices. These interactions play a crucial role in ensuring survival of healthy plants, soil and microbial communities. Many platforms have been developed over the years to isolate these highly complex interactions however, these are designed for small model plants. This creates a need for complementary devices for larger plants, such as sorghum.

**Methods:**

This work introduces a novel platform, EcoFAB 3.0, which is designed to enable studying bioenergy plants such as sorghum for up to 4 weeks in a controlled sterile environment. Several other advantages of this platform such as dark root chambers and user-friendly assembly are also discussed in this work.

**Results and discussion:**

EcoFAB 3.0 was found to replicate previous greenhouse and field observations when comparing an engineered sorghum line overproducing 4-hydroxybenzoic acid (4-HBA) and wildtype (variety BTx430). Consistent with greenhouse and field observations, it was found that the engineered line of sorghum grown in EcoFAB 3.0 had a higher 4-HBA content and a lower dry biomass.

## Introduction

The network of different biochemical processes operating in the rhizosphere is complex. Key factors include soil microbial communities and their metabolic activities, plant-microbe interactions (Berendsen et al., 2012; Qu et al., 2020), soil structure and contents (Bünemann et al., 2018; Karlen et al., 2019), among others. Characterizing and ideally decoupling these processes is a crucial step towards understanding the complex inner-workings of the rhizosphere. Various platforms such as EcoFABs (Gao et al., 2018; Zengler et al., 2019), RootArray (Busch et al., 2012), RootChip (Grossmann et al., 2011, 2012), Tracking Root Interaction System (TRIS) (Massalha et al., 2017), FlowPot (Kremer et al., 2018, 2021) and many others (Sanati Nezhad, 2014; Stanley et al., 2016; Aleklett et al., 2018; Aufrecht et al., 2018; Millet et al., 2019; Ugolini et al., 2024) have been developed to isolate and study these systems. Joelle et al. show reproducible plant traits, as an effect of phosphate starvation, using the EcoFAB 2.0 platform (Sasse et al., 2019). Recently, Novak et al. use EcoFAB 2.0 to show nitrogen starvation modulates root exudation (Novak et al., 2024). A two-channel adaptation of the RootChip, called dual-flow-RootChip, is used by Stanley et al. to show how roots adapt to heterogeneous environments. Root hair were seen to grow asymmetrically in response to asymmetric phosphate perfusion (Stanley et al., 2018). Other studies (Nichols et al., 2010; Hong et al., 2012; Jeong et al., 2015; Mafla-Endara et al., 2021) demonstrate the utility of microfluidic platforms in culturing microbes and studying their interactions in controlled environments. However, these platforms are designed for and compatible with small model plants such as *Brachypodium distachyon* and *Arabidopsis thaliana*. Given that these model plants are typically not used as part of field studies, there is limited ability to compare results. This is because model plants are easy to manipulate, have a short life cycle, and have a relatively small genome size which makes them ideal for quick laboratory testing. *A. thaliana* has been one of the most popular model organisms for plant research over several decades. However, it’s also desirable to have systems for studying sorghum, maize, wheat, and others. There is a need for a complementary platform that provides a controlled environment to grow and study these economically important crops which are significantly larger in size than the model plants.

In this work, we introduce EcoFAB 3.0, a portable and sterile platform designed for studying sorghum for up to four weeks. EcoFAB 3.0 enables rhizosphere imaging, root exudate/leachate collection and has several multi-purpose ports to monitor gaseous exchange, moisture, and temperature among other parameters under sterile conditions. To test the ability of the EcoFAB 3.0 to produce results comparable to field and greenhouse studies, we followed previously published work which characterized engineered sorghum lines accumulating 4-hydroxybenzoic acid (4-HBA) (Lin et al., 2022). 4-HBA is primarily known for manufacturing paraben, which is a widely used preservative in pharmaceutical and cosmetic industries. 4-HBA is a precursor of many valuable products such as deep eutectic solvents (Wang et al., 2018, 2021), Vectran™ (Magalhães et al., 2020). It also has various biological properties such as anti-microbial, anti-inflammatory and others (Chaudhary et al., 2013). In this study, we grow one of the previously characterized engineered 4-HBA sorghum lines in the EcoFAB 3.0 as well as in pots, as a control, and compare 4-HBA production and phenotypic data. The following sections discuss EcoFAB 3.0 fabrication processes, plant growth and harvesting procedure, and comparison with previously published observations.

## Materials and methods

**Figure 1:**
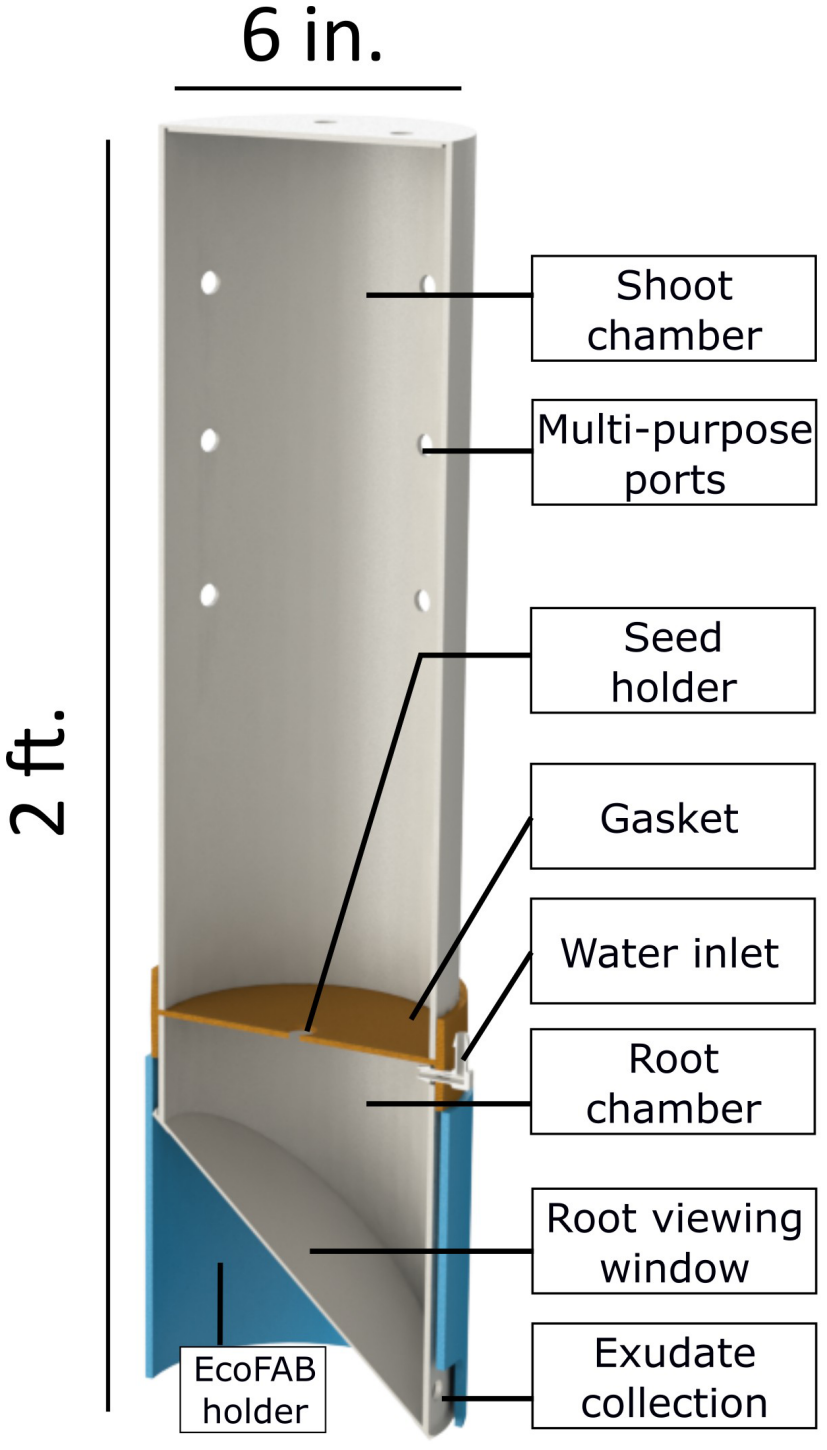
Full EcoFAB 3.0 assembly in section-view.

EcoFAB 3.0 (**Figure 1** below) is fabricated using a combination of custom-made and commercially available parts. The following sub-sections provide details about the different processes employed.

### Machining

**Figure 2:**
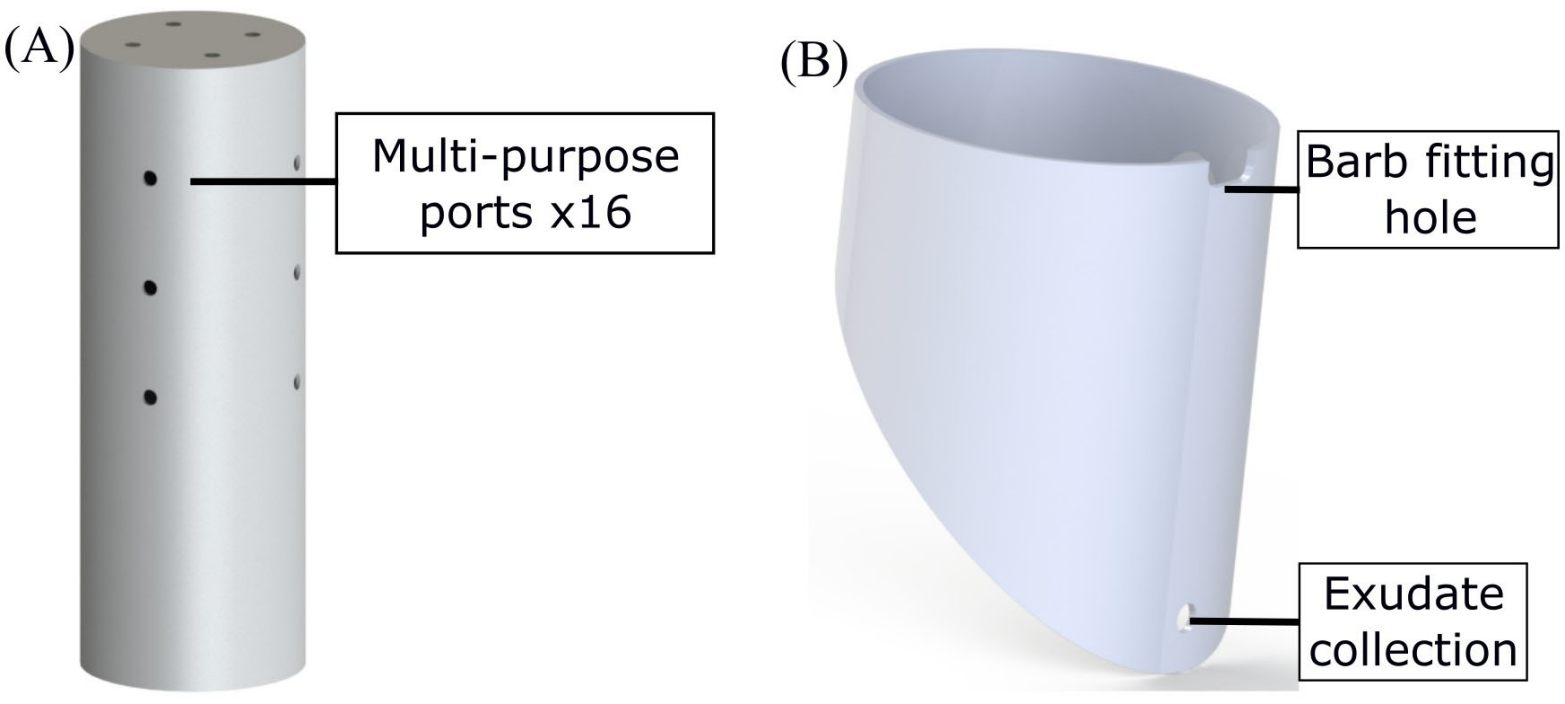
Models of **(A)** shoot and **(B)** root compartments of EcoFAB 3.0.

The main body of the EcoFAB 3.0 comprises of two chambers, one for the shoot (**Figure 2A**) and other for the roots (**Figure 2B**). Both of these chambers are made by machining a 6-inch (OD) clear polycarbonate (PC) plastic pipe (MSC Industrial Supply Co.). The shoot chamber is a 17 inch tall piece of the polycarbonate (PC) pipe with a circular disk glued (high temperature epoxy such as Loctite E-120HP) on top as a lid. The lid is machined out of a PC sheet (McMaster Carr) of a grade comparable to the 6 inch tube. The shoot chamber has holes drilled in the top lid (x4) and the side (x12) walls. These multi-purpose holes are used for ventilation, collecting gases (CO_2_, CH_4_ etc.), and as ports for introducing various sensors (temperature, moisture etc.) in the system.

The root chamber is a 7 inch tall piece of the PC tube with a wedge cut out of one side. This wedge interfaces with a window which is used to view roots grow inside the EcoFAB similar to a rhizotron window (Huck and Taylor, 1982; Taylor et al., 1990). The root chamber has a hole drilled near the bottom for collecting root exudates/leachates. A rectangular slot is also machined out of the top of the root chamber directly above the exudate collection hole. This slot interfaces with two barb fittings which are used to add water/growth medium, vent and collect gases from the root chamber. The whole assembly is seated in an opaque holder (blue part in **Figure 1**) which blocks light from the entire root chamber when in place. The holder is made out of an opaque PVC pipe and has a square slot cut out of the bottom which aligns with the exudate collection tap.

### Injection molding

**Figure 3:**
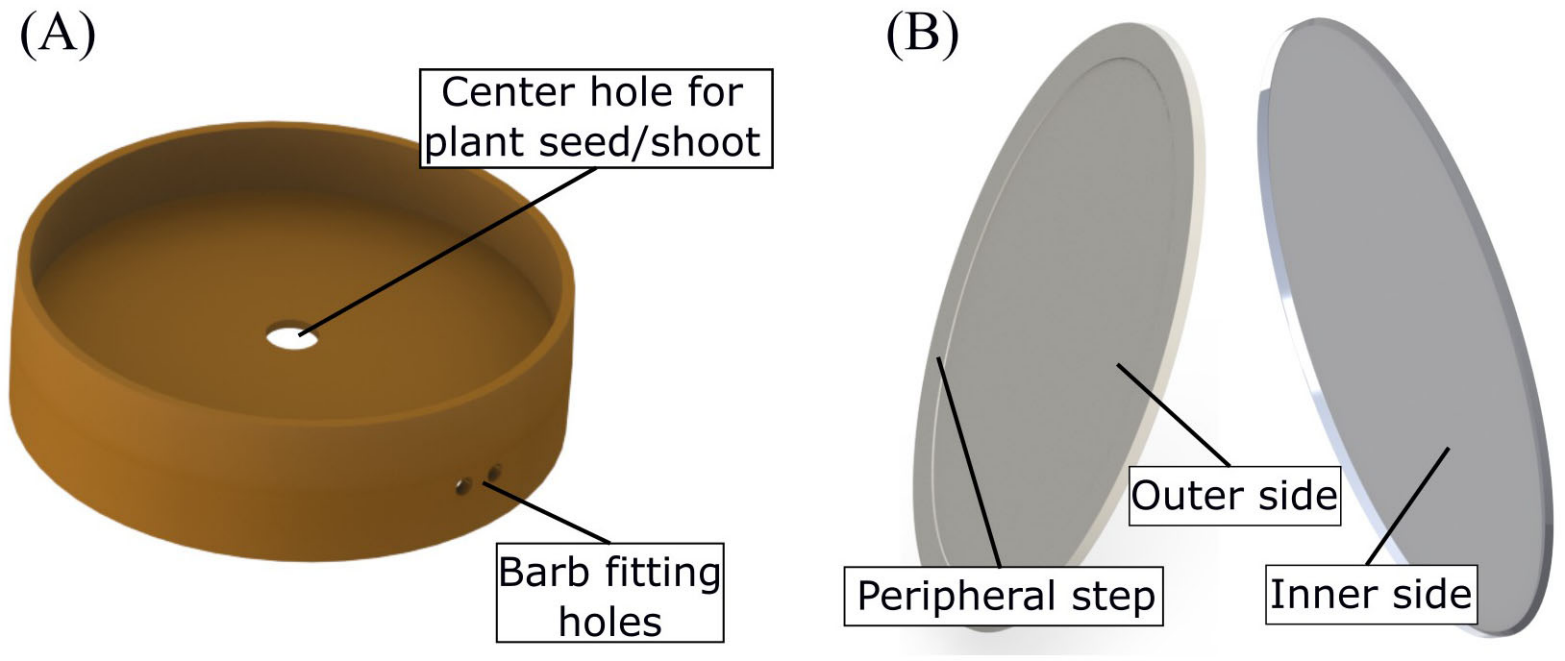
**(A)** Injection-molded silicone gasket which holds the shoot and the root compartments together. **(B)** Two sides of the injection-molded viewing window. The outer side, which forms the root imaging plane, has a peripheral ring-shaped step to prevent damage on the window. The inner side, which interfaces with the root chamber, has a lip which is filled with a silicone sealant.

EcoFAB 3.0 has two injection molded parts: the first is a coupling gasket and the second is the root viewing window. The gasket (**Figure 3A**) holds the two chambers together while isolating them. It has a cylindrical body with a circular disk in the middle. This disk separates the two chambers and has a hole in its center. A plant seed is placed in this hole so that the roots and the shoot grow in their respective chambers. The gasket further consists of two holes on the cylindrical side-wall which interface with the rectangular slot cut into the root chamber. These holes are used to fit the barbs as discussed earlier. It is made using autoclave-compatible black liquid silicone rubber Elastosil 3003/50 A/B with non-cosmetic surface finish on the molds.

The second injection molded part is the root viewing window (**Figure 3B** shows both sides of the window). It is an oval shaped disk, also made of polycarbonate, glued to the root chamber to enable root imaging. It is placed at an angle with respect to the sidewalls to encourage the roots grow along the imaging plane. The window is bonded using solvents such as acetone or chloroform to prevent interference from epoxies or commercial adhesives in metabolomics studies. The roots can be imaged by placing the EcoFAB on a photo scanner (or a microscope) such that the viewing window coincides with the imaging plane. It has a 0.5 mm deep ring-shaped step along the periphery, on the outer side, to prevent damage on the imaging surface when the EcoFAB is placed on the photo scanner. It also has a triangular lip on the inner side that creates a cavity when interfaced with the root chamber, which is filled with a silicone sealant (Momentive/GE RTV102) to create a leak-proof joint. To ensure autoclave compatibility, it is made of clear polycarbonate (Lexan HP1-1H112).

### Commercial parts

EcoFAB 3.0 uses several commercially available parts. All the multi-purpose holes are made leak-proof using high-temperature silicone grommets (McMaster Carr 1061T25). The root exudate collection tap is made using a high-temperature silicone tubing (McMaster Carr 5054K323) operated with a pinch valve (McMaster Carr 5031K12). The water/growth medium ports in the root chamber (as explained above) are made using 90° elbow barb tube fittings (McMaster Carr 5117K76). The holes, if not in use, are blocked using a tapered plug (McMaster Carr 40025K51) or sterile permeable tape (3M Surgical Micropore Tape) to create a breathable vent in the shoot chamber. The coupling gasket is attached to the two chambers using two 6-inch hose clamps (Powertec 70250).

### Growing and harvesting sorghum using EcoFAB 3.0

Engineered sorghum plants overproducing 4-HBA were grown in EcoFAB 3.0 together with the wild-type segregant control (Lin et al., 2022). T3 seeds from Eng-2 line were germinated on a petri dish for four days in a growth chamber and transferred into EcoFAB 3.0 on day five. Growing conditions were 270 µmol/m^2^/s, 27°C, 60% humidity and 16 h of light per day. Plants were kept in the same chamber for another 21 days before harvest. To evaluate the effect of the EcoFAB enclosure on the plant’s immediate environmental conditions, temperature and relative humidity were measured using a SensorPush HT.w Smart Sensor and light intensity was measured using an Onset HOBO MX2202 sensor. The conditions inside the shoot chamber were measured in presence of a 7-day old Eng-2 line sorghum plant over 13 days, followed by measurements outside over 8 days. To compare growth conditions in EcoFAB 3.0 and regular planting pots, one set of five-day-old seedlings from both engineered line and WT were transferred into 2-quart planting pots and grown together next to the EcoFAB 3.0 units inside the same growth chamber. Plants grown in both EcoFAB 3.0 and pots were watered with the same nutrient solution (1/4 tsp. of all-purpose Miracle Gro plant food per liter of water). At each watering, 20 ml of nutrient solution was injected into EcoFAB 3.0 using a syringe connected to the watering port. The plants in EcoFAB 3.0 were watered every seven days.

**Figure 4:**
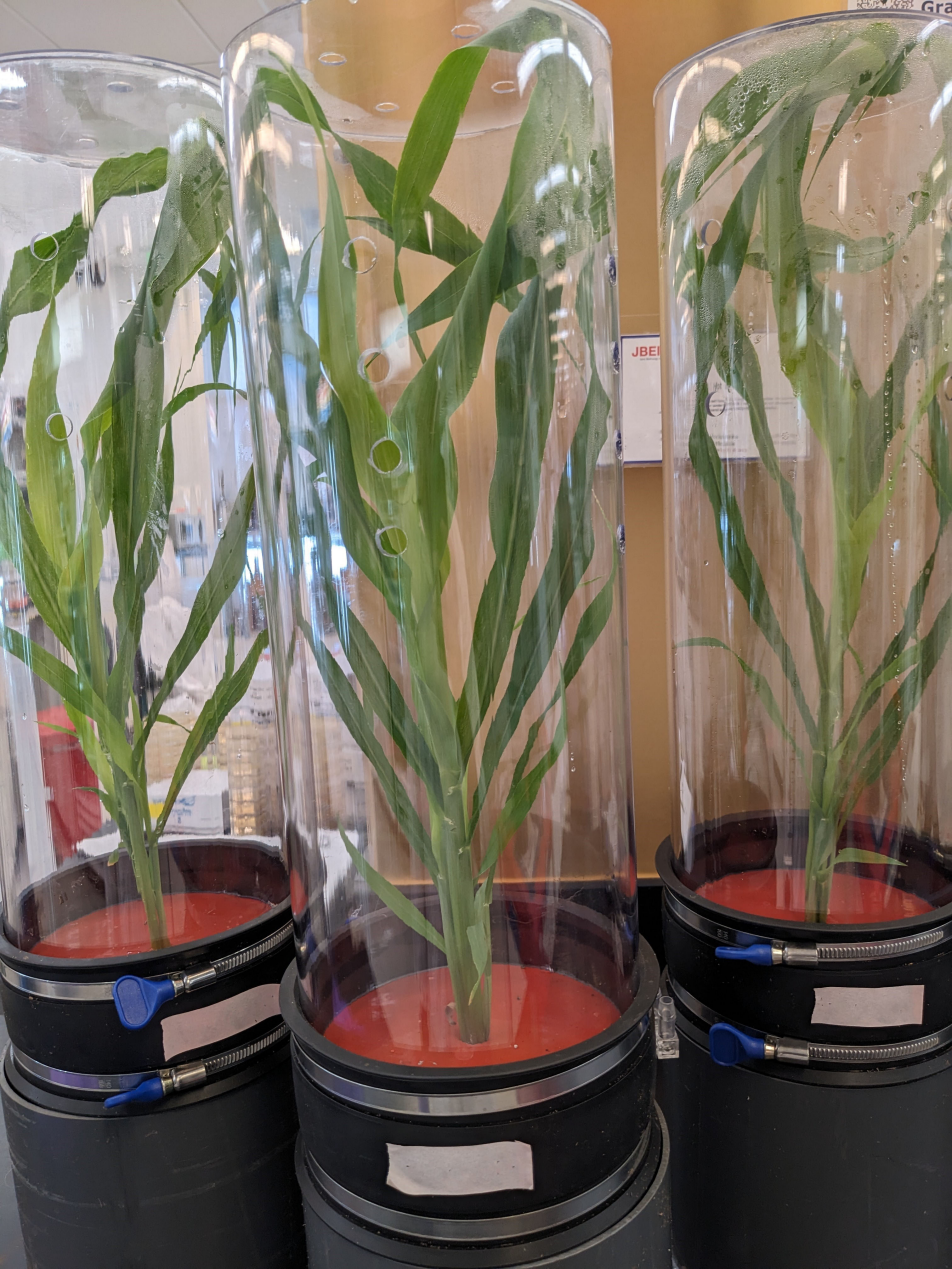
26-day-old sorghum plants in EcoFAB 3.0 before harvest.

Plants were harvested when they were 26 days old (**Figure 4**). Root and shoot chambers were disconnected, and plants were gently removed with the soil still adhering to the roots. The shoot was cut at the crown and weighed for fresh biomass. Shoot tissue was then frozen in liquid nitrogen and lyophilized prior to dry biomass measurements. Shoot length, number of leaves, and tillers were also counted for phenotypic comparison.

### 4-hydroxybenzoic acid analysis

Metabolites in shoot tissues were sequentially extracted from 30 mg dry biomass using 80% methanol (3 x 15 min), followed by acid hydrolysis of the extracts and ethyl acetate partitioning as described by Eudes et al. (2012). Hydrolyzed extracts were reconstituted in 50% methanol for high-performance liquid chromatography (HPLC) analysis as previously described by Rodriguez et al. (2019). In brief, 4-HBA was separated on an Eclipse Plus Phenyl-hexyl column (250 mm length, 4.6 mm diameter, 5 µm particle size; Agilent Technologies, USA) that was maintained at 50°C. 4-HBA was detected by Diode array detectors at 254 nm wavelengths.

## Results and discussion

### EcoFAB 3.0 design, fabrication, and operation

EcoFAB 3.0 design is primarily influenced by the need for a user-friendly, affordable, portable, easy to fabricate, and reusable platform to grow bioenergy crops, such as sorghum, in a sterile environment. The device requires only two hand-operated clamps to tightly seal the shoot and the root chambers with the gasket. Additionally, using autoclave compatible silicone grommets, plugs, and barb fittings to seal/use the multi-purpose ports makes the operation of EcoFAB 3.0 highly user-friendly. It is sized to house economically relevant crops however, it is small enough to be portable and compatible with laboratory growth chambers. The shoot chamber is sized at 17 inches tall based on the average height of 4-week old wheatland sorghum. The root chamber is sized at 7 inches deep to create an approximately 2-quart chamber comparable with pots and to keep the total device height at 2 ft. enabling two shelves of EcoFAB 3.0 to be grown in a standard growth chamber. As these chambers are machined out of stock plastic pipes, the device can be modified to house plants of different sizes by cutting a longer or a smaller section. However, this will affect the number of devices that can be accommodated in a standard growth chamber. The polycarbonate grade used is transparent and stable at 121°C (250°F) which enables imaging, ensures compatibility with autoclaves, and makes the platform reusable. Although the study in this work did not require sterile growth conditions, the EcoFABs were still autoclaved at 121°C, 15 psig for 20 minutes. The sterility of the EcoFABs was confirmed and the relevant details can be found in the **Supplementary Section S1**. The device is designed to maintain many capabilities from currently used platforms such as root imaging and exudate collection. The root viewing/imaging window was injection molded with an SPI A1-A2 finish to ensure microscope compatible optical clarity. The transparent root chamber of the EcoFAB enables monitoring the soil moisture all the way to the bottom of the container. The plants growing in the pots were watered every three days with a larger amount as compared to those in the EcoFABs, which were watered weekly. This difference is attributed to higher humidity levels within the EcoFAB 3.0 device as discussed below.

EcoFAB 3.0 presents several advancements compared to EcoFAB 1.0, 2.0 and other devices. Most importantly, these earlier devices are only suitable for growth of very small plants (<5cm tall) whereas the new device supports plants up to 43 cm tall. Earlier devices also confine root growth to a horizontal plane whereas the new device allows roots to explore a much larger soil volume (2 liters) without this constraint. EcoFAB 3.0 employs highly user-friendly fabrication and assembly processes (as described in the Materials and Methods section) in contrast to the other platforms which require specialized fabrication techniques such as polydimethylsiloxane (PDMS)-based casting or laborious assembly. This further enhances the flexibility of the size of the whole device as a user can customize each compartment’s size by machining a smaller (or larger) piece of the polycarbonate tube to accommodate different plants.

Average temperature and light intensity (27.8°C and 6175 lux) measured inside the EcoFAB follow the conditions measured outside (27.3°C and 6529 lux) closely. Average relative humidity measured outside the EcoFAB also remains steady at 64%. However, the average relative humidity inside the shoot chamber increases to 77% over 13 days due to the growing plant. Thus, we recommend increasing the size and number of the ventilation holes to better regulate humidity in future studies. **Supplementary Section S2** shows detailed measurements (**Supplementary Figure S2**) of temperature, light intensity and relative humidity. Overall, we found that EcoFAB 3.0 supports robust growth of sorghum and requires less plant maintenance. Roots were imaged successfully using a photo scanner. **Supplementary Section S3** shows image time series of sorghum growth in an EcoFAB 3.0 (**Supplementary Figure S3**) and a 2-quart pot (**Supplementary Figure S4**). It also highlights the root imaging capabilities of EcoFAB 3.0 through a time-lapse in **Supplementary Figure S5** and **Supplementary Video S1**.

### Phenotypic parameters and 4-HBA analysis

Engineered sorghum (4-HBA line) harvested from EcoFAB 3.0 showed similar dry biomass compared to those grown in pots. However, the wildtype showed 12% less dry biomass in EcoFABs as compared with that collected from the pots. In both EcoFAB 3.0 and pots, the 4-HBA line showed reduced biomass than WT (16% and 14% respectively). This observation is consistent with the biomass data collected from the field growth conditions (4-HBA line showed 11% biomass yield reduction) (Lin et al., 2022). We attribute the lack of statistical significance in reduction of plant biomass in the EcoFABs to the smaller sample size (3 replicates for WT) in this work compared with previous studies which had four replicated 18 m^2^ plots consisting of four rows and ~355 plants each (~1420 plants total). It should be noted that although the plants harvested from the field (4-month-old) were much older than those harvested from the EcoFABs, the proportion of biomass reduction was still found to be consistent. Both the WT and 4-HBA line plants grown in the greenhouse were taller than those grown in the growth chamber. This difference is accredited to the larger intensity of the natural light source and poorer control on the temperature and relative humidity in a greenhouse. Unlike the wheatland sorghum we found that the BTx430 plants reached the top of the shoot chamber in 26 days. Crowded leaves with reduced effective area available for photosynthesis can result in shorter shoots and lower 4-HBA synthesis. It is recommended that future studies with this plant line either restrict the experiment duration to approximately 3 weeks or use 3-4 inches taller shoot chambers. However, the latter will reduce the number of devices that can fit within a standard growth chamber, as discussed earlier. Number of leaves and tillers, however, were found to be consistent in all the growth conditions.

**Figure 5:**
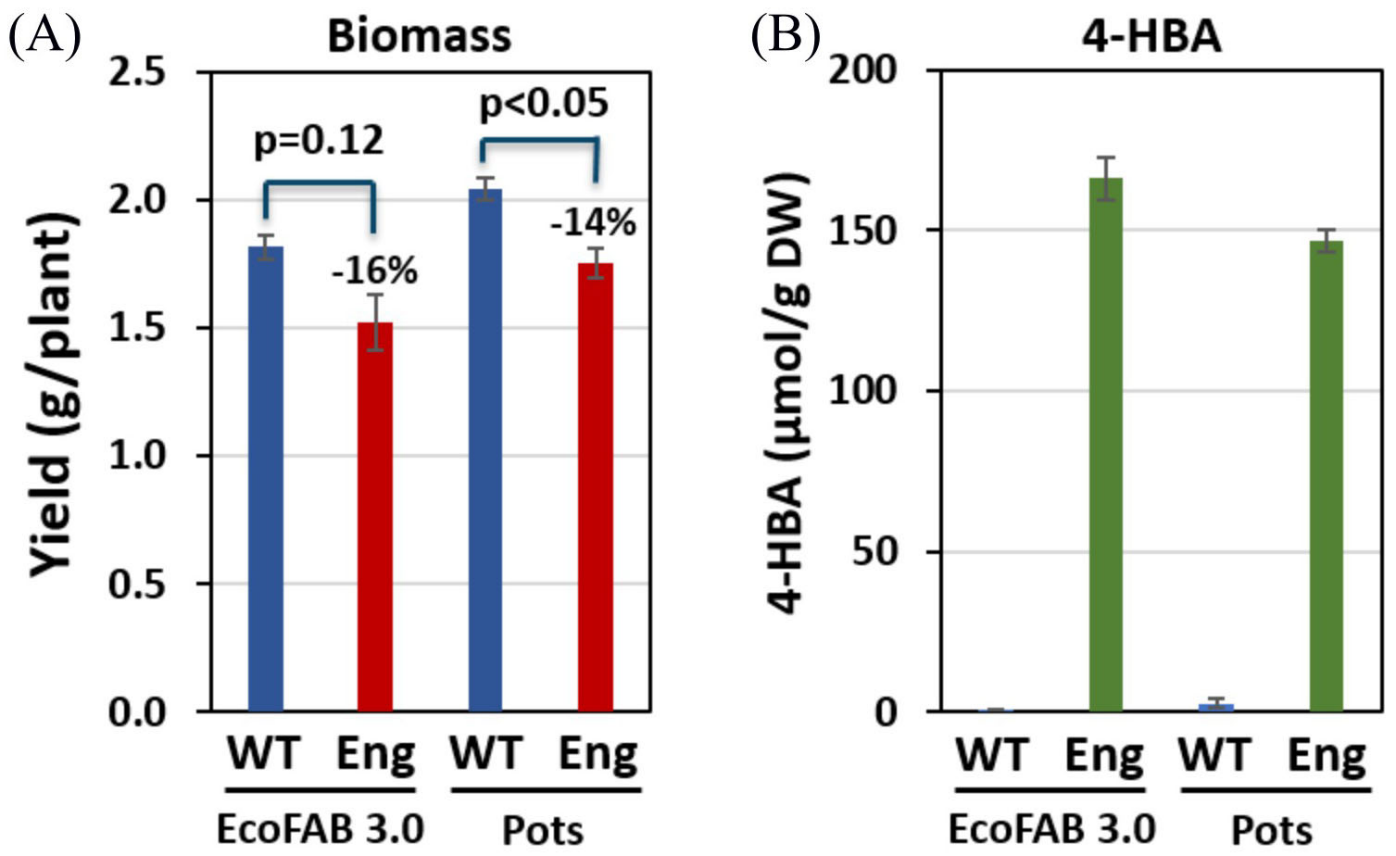
Biomass yield and 4-HBA content in engineered (Eng) and wild-type (WT) sorghum **(A)** Engineered sorghum shows a 16% and 14% reduction in biomass in comparison to the wildtype grown in EcoFAB 3.0 and pots, respectively. **(B)** Engineered sorghum shows two orders of magnitude increase in 4-HBA content as compared to the wildtype.

4-HBA contents measured in the plants grown in EcoFAB 3.0 were not significantly different from those measured in the same plant genotypes grown in the pots. 4-HBA concentration in the engineered line was significantly higher than that in WT, which aligns with the data collected from greenhouse-grown plants (Lin et al., 2022). Moreover, 4-HBA content in the engineered line (166 µmol/g dry wt.) is comparable to that measured in greenhouse-grown engineered plants at the same age (~1-month-old) (**Supplementary Figure S6**). **Table 1** below summarizes the comparison of observations between EcoFABs, pots, and greenhouse. **Figure 5** shows the biomass and 4-HBA contents measured in plants grown in EcoFAB 3.0 and pots in the growth chamber.

**Table 1:** Comparison of 4-HBA content and phenotypic parameters between engineered sorghum (Eng) and wild-type control (WT) grown in EcoFAB 3.0 (WT: n=3 and Eng: n=4), pots (n=4) and greenhouse (WT: n=5 and Eng: n=4).

	EcoFAB 3.0		Pots		Greenhouse	
	WT	Eng	WT	Eng	WT	Eng
4-HBA (µmol/g dry wt.)	0.75 ± 0.15	166.07 ± 6.85	2.57 ± 1.39	146.85 ± 3.49	11.52 ± 1.00 (Lin et al., 2022)	162.90 ± 5.80 (Lin et al., 2022)
Shoot length (inch)	28.62 ± 0.62	26.97 ± 0.16	27.25 ± 0.39	25.44 ± 0.85	31 ± 0.55	31 ± 0.46
Number of leaves	7	6	6	6	7	7
Number of tillers	2	3	3	3	2	3

While these results are encouraging, many more studies would need to be done to validate EcoFAB 3.0’s translatability to greenhouse and to fields. It will be important to perform additional studies comparing the performance of this device in supporting analysis of soil microbial communities. This design also has some limitations that should be pointed out. Firstly, due to its big size, it is challenging to assemble inside a bio-safety hood which is necessary for sterile conditions. Additionally, although the root viewing window is compatible with microscopy, its shape and size does not fit on standard microscope stages. Hence a custom stage or an adapter to a standard stage is needed to fit EcoFAB 3.0 on a microscope. We anticipate that EcoFAB 3.0 should be extensible to a number of other plants, especially those with similar phenology to sorghum (e.g. maize).

## Conclusion

This work presents a new device, EcoFAB 3.0, for studying sorghum in a controlled sterile environment for up to 4 weeks. EcoFAB 3.0 supports more naturally growing roots in a dark large 2-liter root chamber. It has several multi-purpose ports that can be used for exudate collection, ventilation, and introducing sensors to monitor gaseous exchange, temperature and other parameters. It features a rhizotron-like window which enables capturing time-lapse images of roots using a photo scanner or an optical microscope. In this work, we demonstrate EcoFAB 3.0 is able to produce observations comparable to those found in field and greenhouse studies. Sorghum plants were successfully grown past the five-leaf stage in EcoFAB 3.0 for 26 days before harvest. An engineered line was compared with the wildtype for its biomass and 4-hydroxybenzoic acid (4-HBA) accumulation. Consistent with the greenhouse observations, the engineered line grown in the EcoFABs maintained a drastic increase in 4-HBA accumulation (166.07 ± 6.85 µmol/g dry wt.). Additionally, in agreement with field results, the engineered line showed a 16% reduction in the biomass. Although it should be noted that the field-grown plants used as reference were older than those used in this work.

## Data Availability

The original contributions presented in the study are included in the article/**Supplementary Material**. Further inquiries can be directed to the corresponding author.
