# Supplementary material for: EcoFAB 3.0: a sterile system for studying sorghum that replicates previous field and greenhouse observations
Source: Front Plant Sci. 2024 Oct 7;15:1440728. doi: 10.3389/fpls.2024.1440728 (PMC11491363; doi:10.3389/fpls.2024.1440728)
Supplement: Supplementary file 1 [file DataSheet1.pdf]

EcoFAB 3.0: A sterile system for studying sorghum that replicates previous field and greenhouse observations

Supplementary Information

Kshitiz Gupta[1,2], Yang Tian[3,4], Aymerick Eudes[3,4], Henrik V. Scheller[3,4], Anup K. Singh[1,2], Paul D. Adams[1,4], Peter F. Andeer[4], and Trent R. Northen[1,4,*]

[1]Technology Division, Joint BioEnergy Institute, Emeryville, CA, USA

[2]Engineering Directorate, Lawrence Livermore National Laboratory, Livermore, CA, USA

[3]Feedstocks Division, Joint BioEnergy Institute, Emeryville, CA, USA

[4]Environmental Genomics and Systems Biology, Lawrence Berkeley National Laboratory, Berkeley, CA, USA

*Corresponding Author: Trent R. Northen (trnorthen@lbl.gov)

Section S1: Sterilizing EcoFAB 3.0

EcoFAB 3.0 is made using autoclave compatible materials. All the parts should be thoroughly rinsed with DI water before autoclaving. Gravity displacement cycle is used to sterilize EcoFAB 3.0 at 121°C and 15 psig for 20 min followed by 30 min of drying time. An alternative sterilizing strategy can be used in which the parts are thoroughly rinsed with ethanol and kept in a bio-safety cabinet under UV light for an hour.

To test the sterility, LB medium was poured in the autoclaved parts and collected in two autoclaved glass bottles. One bottle was stored at room temperature and the other at 30°C. Both samples were accompanied by negative controls which were prepared by collecting LB medium in sterile bottles but without exposing them to the EcoFAB parts. The EcoFAB was assembled with the leftover medium in the root chamber and stored at 37°C. A conical tube with 40 ml LB medium was placed without a cap in the hole for plant seeds in the gasket to test sterility of the shoot compartment. After a week on shelves, all the bottles were incubated for a week on a shaker at 200 rpm and 37°C. No microbial growth was observed in the EcoFAB and any of the bottled samples after 17 weeks. 50 µl of LB medium from the root and the shoot chambers, after 17 weeks, was plated in LB agar petri dishes (2 replicates). The EcoFAB was also re-assembled with an open LB agar plate in the shoot chamber. No microbial growth was observed in the agar plates after incubation at 30°C for 3 days. Figure S1 below shows the collected samples, the controls, the assembled EcoFAB 3.0, and the LB agar plates after incubation.

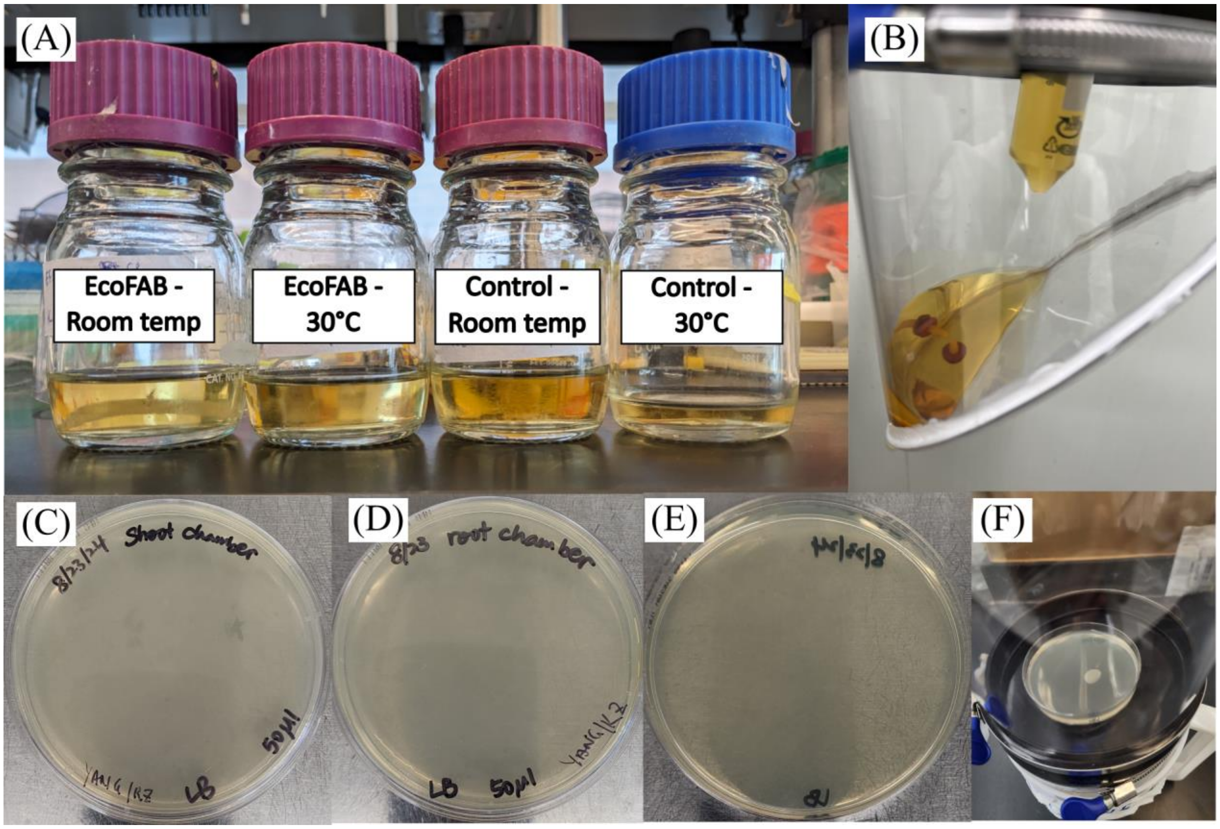

Figure S1: Sterility test for autoclaved EcoFAB 3.0. (A) LB medium collected from autoclaved EcoFAB 3.0 parts along with negative controls after 17 weeks of incubation. (B) EcoFAB 3.0 assembled with LB medium show no microbial growth after 17 weeks of incubation. (C and D) LB agar petri dishes plated with LB medium collected from the shoot and the root chambers and (E and F) the open LB agar petri dish incubated inside the EcoFAB for 3 days at 30°C show no microbial growth.

Section S2: Temperature, light intensity, and relative humidity measurement comparison inside and outside an EcoFAB 3.0

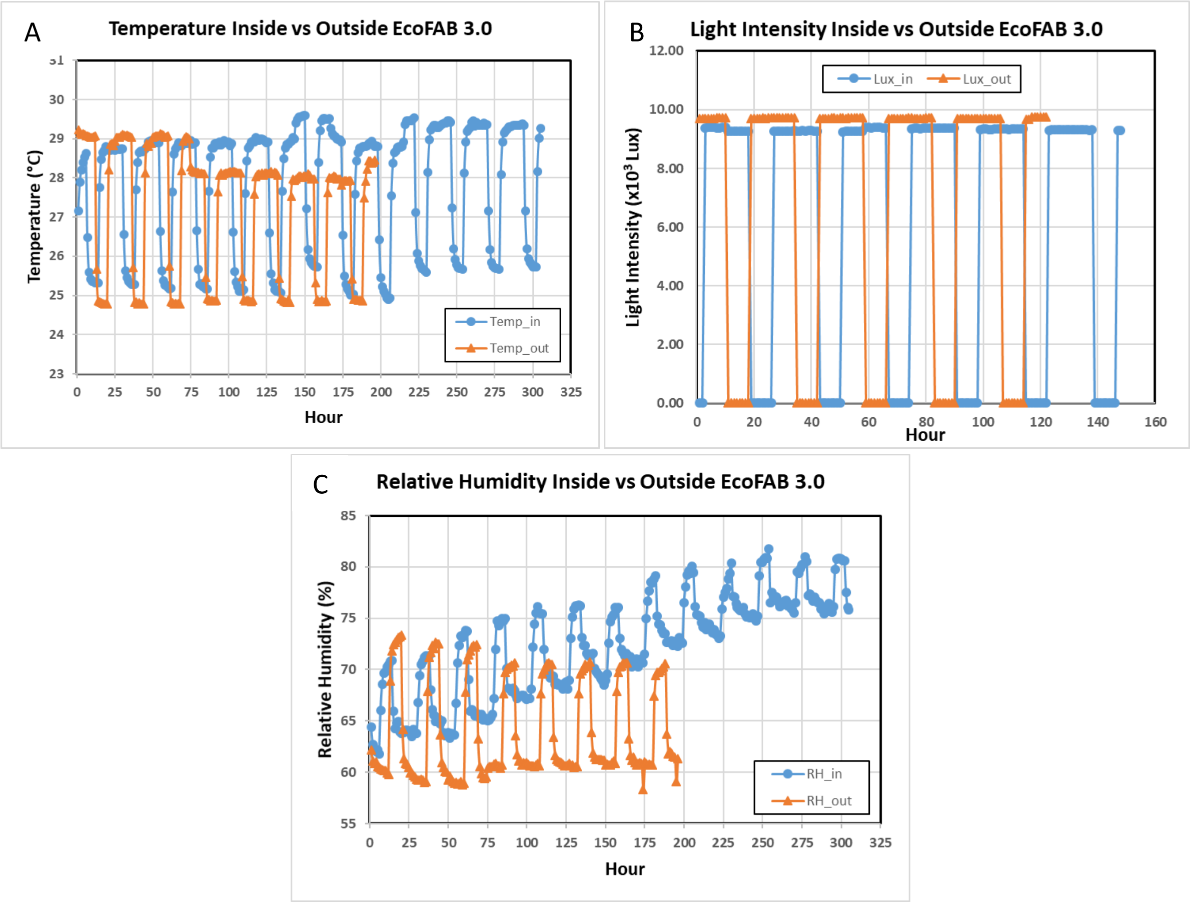

Figure S2: Temperature (A), light intensity (B), and relative humidity (C) measured inside (blue dots) and outside (orange triangles) of an EcoFAB 3.0. Average temperature and light intensity are comparable in both measurements and remain stable as the plant grows. Average relative humidity inside the EcoFAB 3.0 increases with time, as the plant grows.

Section S3: Growing sorghum in EcoFAB 3.0 and root time-lapse imaging

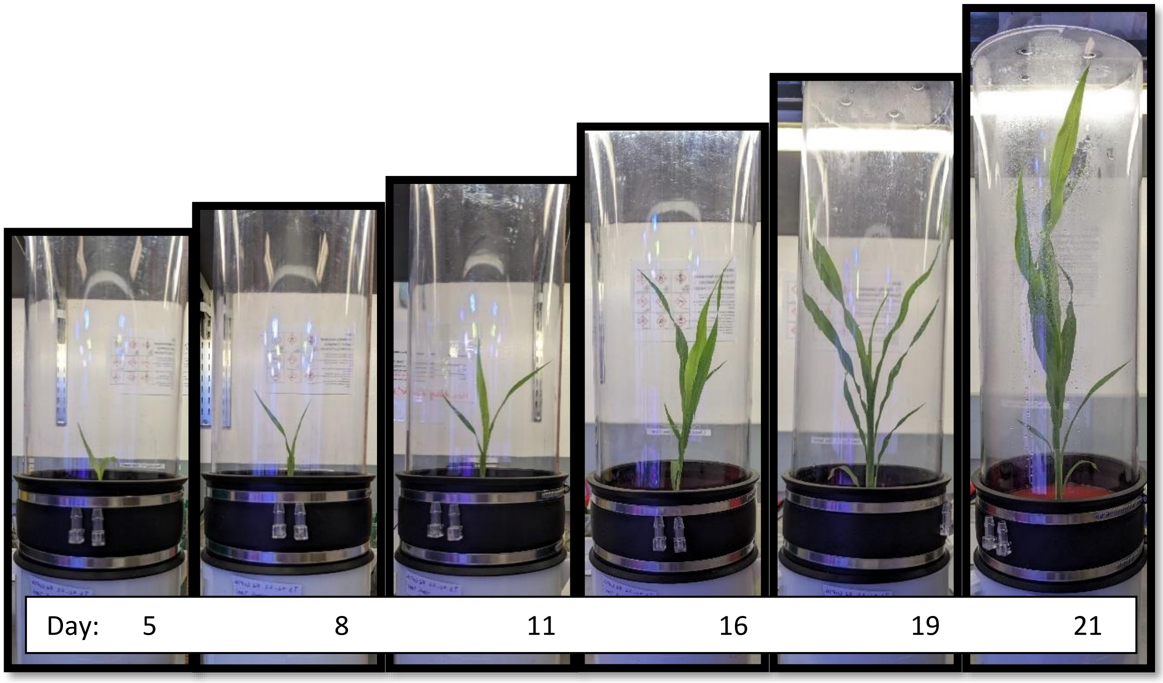

Figure S3: Growing sorghum in EcoFAB 3.0.

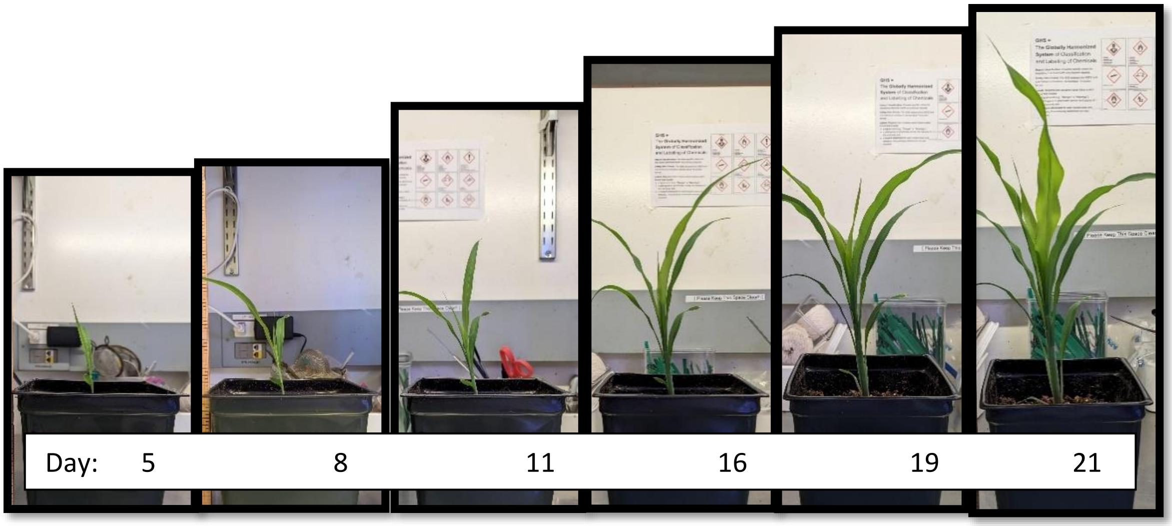

Figure S4: Growing sorghum in a 2-quart pot as a reference.

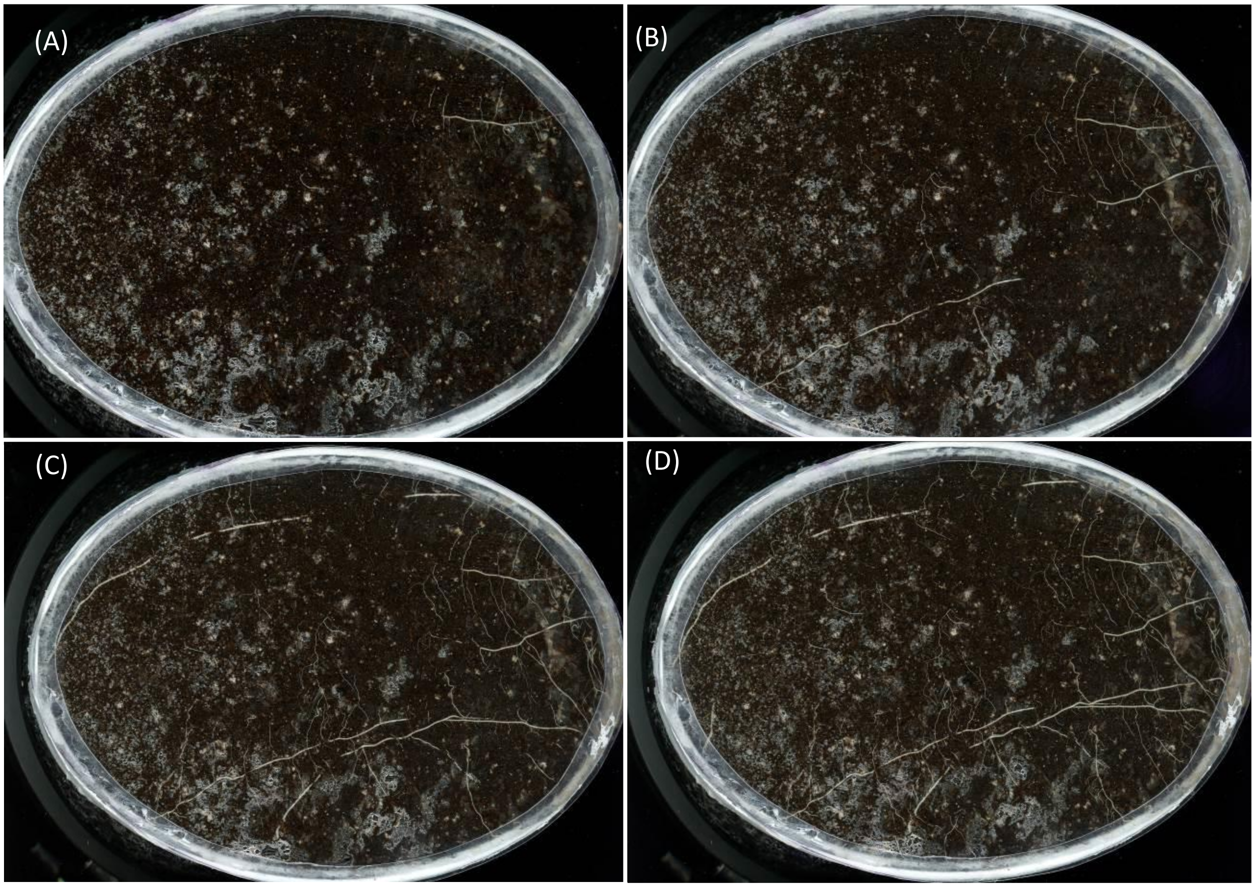

Figure S5: Root images captured through the injection molded viewing window on day 11 (A), 16 (B), 19 (C), and 21 (D) using a photo scanner.

Section S4: 4-hydroxybenzoic acid content comparison between engineered sorghum grown in EcoFAB 3.0 and greenhouse.

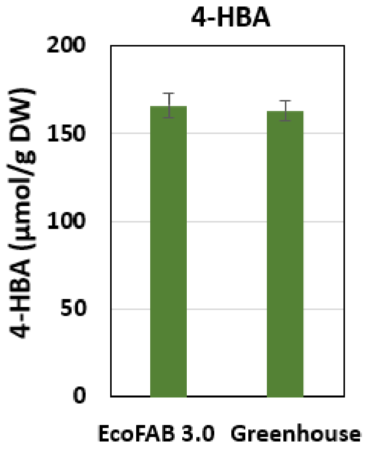

Figure S6: 4-HBA content in ~1-month old engineered line grown in EcoFab 3.0 and greenhouse
